# Molecular Docking and MD Modeling Techniques for the Development of Novel ROS1 Kinase Inhibitors

**DOI:** 10.3390/ph19020229

**Published:** 2026-01-28

**Authors:** Mohammad Jahoor Alam, Arshad Jamal, Shaik Daria Hussain, Shahzaib Ahamad, Dinesh Gupta, Ashanul Haque

**Affiliations:** 1 Department of Biology, College of Science, University of Ha’il, Ha’il 2440, Saudi Arabia; ar.jamal@uoh.edu.sa; 2 Department of Physiotherapy, College of Applied Medical Sciences, University of Ha’il, Ha’il 2440, Saudi Arabia; sd.hussain@uoh.edu.sa; 3 Translational Bioinformatics Group, International Centre for Genetic Engineering and Biotechnology (ICGEB), Aruna Asaf Ali Marg, New Delhi 110067, India; shah.bioinfo@gmail.com (S.A.); dinesh.bioinfo@gmail.com (D.G.); 4 Department of Chemistry, College of Science, University of Ha’il, Ha’il 2440, Saudi Arabia

**Keywords:** ROS1, crizotinib, molecular docking, MM-GBSA, MD simulation

## Abstract

**Background:** Chemotherapy is a cornerstone of cancer treatment; however, resistance to first-line chemotherapeutic agents remains a major challenge. ROS1, one of fifty-eight receptor tyrosine kinases, has been implicated in various cancer subtypes, including glioblastoma, non-small-cell lung cancer, and cholangiocarcinoma. Notably, the Gly2032Arg mutation in the ROS1 protein has been linked to resistance against the kinase inhibitor crizotinib. **Objectives:** Given the challenge, we conducted a comprehensive in silico study to identify new drug candidates. **Methods:** The study starts with modeling the Gly2032Arg-mutated ROS1 protein, followed by structure-based screening of the PubChem database. **Results:** Out of 1760 molecules screened, we selected the top 4 molecules (PubChem CID: **67463531**, **72544946**, **139431449**, and **139431487**) with structural features similar to crizotinib, a high docking score, and drug likeness. To further validate the effectiveness of the identified compounds, we assessed their binding affinity using the Molecular Mechanics with Generalized Born Surface Area (MM-GBSA) scoring method. To underpin the behavior and stability of protein–ligand complexes, 500 ns molecular dynamics (MD) simulations were conducted, and parameters including RMSD, RMSF, and H-bond dynamics were studied and compared. Density functional theory (DFT) at the B3LYP/6-31G* level was performed to elucidate molecular features of the identified compounds. **Conclusions:** Overall, this study sheds light on a new series of compounds effective against mutated targets, thereby offering a new horizon in this area.

## 1. Introduction

Cancer remains the second leading cause of global mortality and morbidity, resulting in nearly 8 million deaths and 14 million new cases each year. A recent study indicates that both the incidence and mortality rates of cancer are expected to increase in the coming decades. Among various forms of cancers, non-small-cell lung cancer (NSCLC) is a prevalent form of lung cancer, constituting about 85% of all lung cancer cases [1,2]. Despite ongoing reports of potential treatments, fundamental challenges persist in this area, including late detection, metastasis, the absence of selective drugs, diverse treatment responses influenced by genetic, epigenetic, or environmental factors, and the almost inevitable emergence of tumour resistance [3,4,5]. Addressing these complexities remains a crucial focus in cancer research. The proto-oncogene receptor tyrosine kinase ROS1 is implicated in various cancers, including NSCLC [6,7,8,9,10]. Aberrations in the ROS1 gene, such as gene fusions, have been identified in a subset of NSCLC cases, leading to constitutive activation of the ROS1 kinase domain [11,12]. In NSCLC, ROS1 rearrangements are considered driver mutations that contribute to uncontrolled cell growth and proliferation [13,14]. Understanding the molecular mechanisms and signalling pathways regulated by ROS1 is essential for developing targeted therapies and advancing precision medicine to manage NSCLC [15,16]. Targeting ROS1 with kinase inhibitors, such as crizotinib, has shown therapeutic efficacy in treating ROS1-positive NSCLC [15,17,18]. However, most patients treated with crizotinib develop resistance within the first year.

Numerous theoretical and experimental studies have been undertaken to identify new drug candidates against ROS1 [19]. For example, Vanajothi et al. [20] studied ROS1 as a crucial biomarker in cancer progression and identified potential inhibitors using structure-based, atom-based, and pharmacophore-based virtual screening. Molecular docking and MM-GBSA calculations results suggested strong binding affinity and favorable ADME properties. However, the effectiveness of the inhibitors against mutated ROS1 remains uncertain. Additionally, 100 ns molecular dynamics (MD) simulation is insufficient to assess protein–ligand behavior. Wu et al. [21] explored the inhibitory mechanism of lorlatinib against WT and G2032R-mutated ROS1 using MD simulations and free energy calculations. They noted that the G2032R mutation reduces lorlatinib’s binding affinity by increasing conformational entropy and decreasing solvation energy. However, like the previous study, the 50 ns MD simulation duration is insufficient for a comprehensive trajectory analysis. Pathak et al. [22] identified potential inhibitors for WT and G2032R-mutated ROS1 using pharmacophore modelling, virtual screening, docking, and MD simulations. Although five compounds showed strong binding and stability, the study lacks longer MD simulations. Similarly, Hasan and co-workers [23] investigated the effects of the G2032R and D2033N mutations in ROS1 using MD simulations. They demonstrated that such mutation induces structural instability and drug resistance. In addition, conformational changes were apparent, especially at the ATP-binding site.

Considering the above-mentioned challenges and in pursuit of identifying new drugs effective in combating cancer resistance, we conducted an extensive in silico study of the Gly2032Arg-mutated ROS1 protein. We used the PDB ID 7Z5X protein as a template (residues 1937–2225) to model the complete structure [24]. The PubChem database was used to identify new molecules with characteristics similar to those of crizotinib, yielding 1760 molecules. Based on their binding free energy, MMGBSA scores, and drug-likeness, the top four candidates, namely PubChem CID: **67463531**, **72544946**, **139431449**, and **139431487**, were then selected. To assess their stability, MD simulations were performed, and root mean square deviation (RMSD), root mean square fluctuation (RMSF), secondary structure elements (SSEs), H-bonding, and protein–ligand interaction results were analyzed. Finally, density functional theory (DFT) calculations were conducted to evaluate molecular properties and descriptors. The results were compared against crizotinib as the reference.

## 2. Results

### 2.1. Homology Modeling and Validation

**Figure 1:**
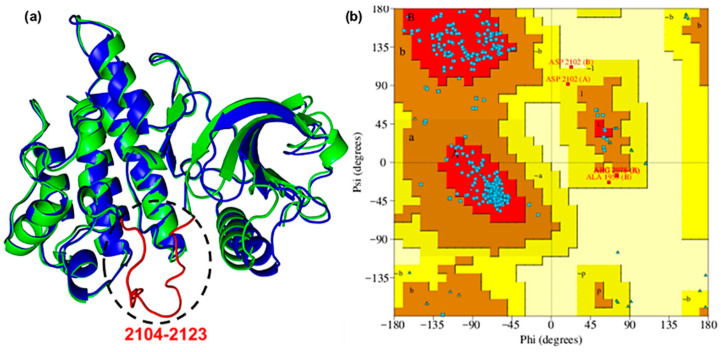
(**a**) Superimposed ROS1 protein (PDB: 7Z5X, blue color) onto the model structure (green color), and the missing residues 21042123 (red loop). (**b**) Ramachandran plot.

To obtain a detailed three-dimensional structure of the ROS1 protein incorporating the missing residues, homology modelling was performed using the Swiss-Model server. The missing segment (2104–2123: GLARDIYKNDYYRKRGEGLL) was modelled using PDB ID 7Z5X (residues 1937–2225) as a template [24,25]. The reconstructed loop was optimized and refined, which was confirmed by a low RMSD (0.71 Å) when aligned with the reference protein (Figure 1a) [26]. The Ramachandran plot revealed that 93.1% of the analyzed regions corresponded to the most favored conformations (Figure 1b). Additionally, 5.8% were allowed, and 0.9% were generously allowed, indicating less optimal but still permissible conformations. Only 0.2% of the plot was found in disallowed regions. Collectively, these findings substantiated the model’s adequacy and reliability [27].

### 2.2. Molecular Docking

**Figure 2:**
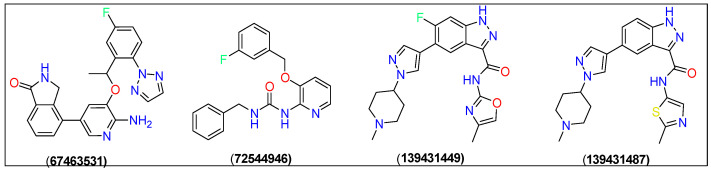
Chemical structures of the top four candidates, namely PubChem CID: **67463531**, **72544946**, **139431449**, and **139431487**.

**Figure 3:**
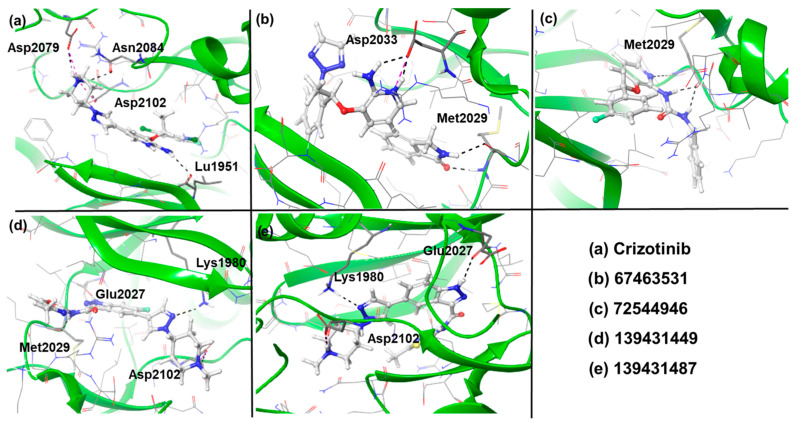
Three-dimensional images of ROS1 with the reference and the identified compounds (**67463531**, **72544946**, **139431449**, and **139431487**). H-bond interactions are shown as black dotted lines.

The modelled protein, as obtained above, was then used as a receptor to identify suitable ligands that bind the protein’s active pocket. Molecular docking was performed using Glide XP, and the results are presented in Table 1 and Figure 2 and Figure 3. A comprehensive search of the PubChem database was conducted to identify potential ROS1 inhibitors, applying key drug-likeness filters such as molecular weight (MW < 500 Da), H-bond donors/acceptors, and logP values. This initial screening yielded 1760 compounds, which were subjected to molecular-docking-based virtual screening. Based on binding affinity, MM-GBSA scores, and drug-likeness properties, the top four promising candidates were shortlisted for further investigation.

Docking studies yielded four new compounds 4-(6-amino-5-(1-(5-fluoro-2-(2H-1,2,3-triazol-2-yl)phenyl)ethoxy)pyridin-3-yl)isoindolin-1-one (**67463531**), 1-benzyl-3-(3-((3-fluorobenzyl)oxy) pyridin-2-yl)urea (**72544946**), 6-fluoro-N-(4-methyloxazol-2-yl)-5-(1-(1-methyl piperidin-4-yl)-1H-pyrazol-4-yl)-1*H*-indazole-3-carboxamide (**139431449**), and 5-(1-(1-methylpiperidin-4-yl)-1H-pyrazol-4-yl)-N-(2-methylthiazol-5-yl)-1H-indazole-3-carboxamide (**139431487**) as the ROS1 inhibitors (Figure 2). Crizotinib was used the reference compound in this study.

**Figure 4:**
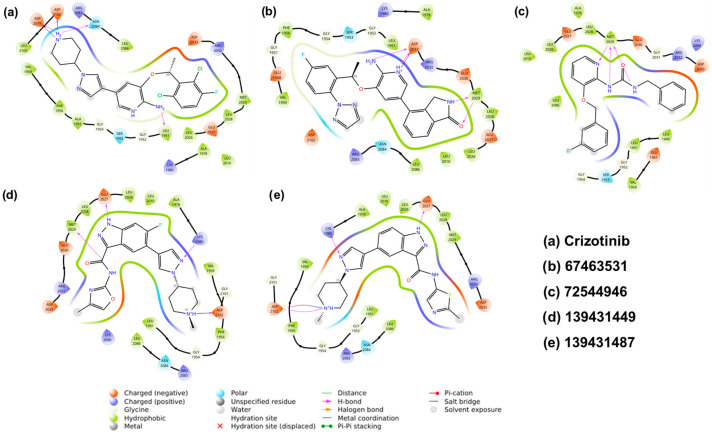
Two-dimensional interactions of ROS1 with the reference and the screened compounds (**67463531**, **72544946**, **139431449**, and **139431487**). H-bond interactions are shown as pink arrows.

Docking results suggest that all the identified compounds displayed higher affinity (XP Gscore = −9.68 to −11.24 kcal/mol) than crizotinib (XP Gscore = −7.50 kcal/mol). The reference compound formed four H-bonds with amino acids Leu1951, Asp2079, Asn2084, and Asp2102 and interacted with residues such as Gly1952, Ser1953, Gly1954, Ala1955, Phe1956, Val1959, Lys1980, Ala1978, Leu2010, Leu2026, Glu2027, Leu2028, Met2029, Arg2032, Asp2033, Arg2083, Leu2086, and Leu2105 through Van der Waals interactions (Figure 3a and Figure 4a). Among the screened compounds, **67463531** showed the lowest XP Gscore (–11.24 kcal/mol) and formed three H-bonds involving Met2029 and Asp2033. Comparing the structure of this compound with the reference molecule, the 2,6-dichloro-3-fluorophenyl and piperidine groups in crizotinib are replaced by 5-fluoro-2-(2H-1,2,3-triazol) and isoindolin-1-one moieties, respectively, while the 2-aminopyridine group remains intact. The latter group (isoindoline-1-one) served as the HBD/HBA fragments, while the triazole ring was found to be exposed to the solvent. Such replacement also modified the way the 2-aminopyridine group interacted with the receptor, which is important when considering the ROS mutation. The ligand was found to be further stabilized by other non-covalent interactions (Figure 3b and Figure 4b). The compound **72544946** exhibited a slightly lower affinity (XP Gscore = −10.63 kcal/mol) and one H-bond (with residue Met2029). However, the two benzyl rings interacted with neighbouring residues, including Leu1949, Leu1951, Gly1952, Ser1953, Gly1954, Val1959, Glu1961, Lys2090, Lys1976, Ala1978, Lys1980, Leu2010, Leu2086, Leu2026, Glu2027, Leu2028, Glu2030, Gly2031, Arg2032, Asp2033, and Thr2036 (Figure 3c and Figure 4c).

The last two compounds, **139431449** and **139431487**, are indazole-based and share a high degree of structural similarity. This also explains why they exhibited very close affinity (−9.77 and −9.68 kcal/mol, respectively). Both compounds formed three H-bonds with Lys1980, Glu2027, and Asp2102, while **139431449** formed an additional interaction with Met2029 (Figure 3d,e and Figure 4d,e). Following the docking studies, we also estimated the binding free energies (ΔG_Bind_) from MM-GBSA calculations. Except for **139431449** (−55.31 kcal/mol), all other compounds displayed the highest negative binding energy (−56.10 to −58.31 kcal/mol) and were better than the reference compound (−55.59 kcal/mol).

### 2.3. Physicochemical and Pharmacokinetic Profiling

ADME/T and pharmacological profiling play a vital role in the drug discovery process. QikProp analysis confirmed that crizotinib and four shortlisted compounds (**67463531**, **72544946**, **139431449**, and **139431487**) met all drug-like features and obeyed Lipinski’s Rule of Five (RO5) (Table 1) [28]. All selected compounds exhibited MW < 500 Da, a polar surface area (PSA) within the acceptable range, and favorable H-bonding fragments (donors: 1–3, acceptors: 3.75–8). Human oral absorption (%HOA) ranged from 77% to 92%, indicating good bioavailability. Additionally, properties such as blood–brain barrier permeability and solubility were within acceptable limits, making these compounds promising candidates for further studies.

The selected compounds were also evaluated for physicochemical and pharmacokinetic properties (Table 1) using the modules available in Qikprop [29]. All compounds exhibited promising permeability across cell membranes (QPPCaco > 88) and moderate brain penetration (QPlogBB between −0.14 and −1.27). Solubility values (QPlogS) ranged from −5.806 to −3.992, indicating good drug-likeness. Notably, all candidates showed acceptable human serum albumin binding (QPlogKhsa) and metabolic stability (metab: 3–6). The overall results suggest that these compounds possess favorable ADME profiles.

### 2.4. MD Simulation

#### 2.4.1. RMSD Analysis

**Figure 5:**
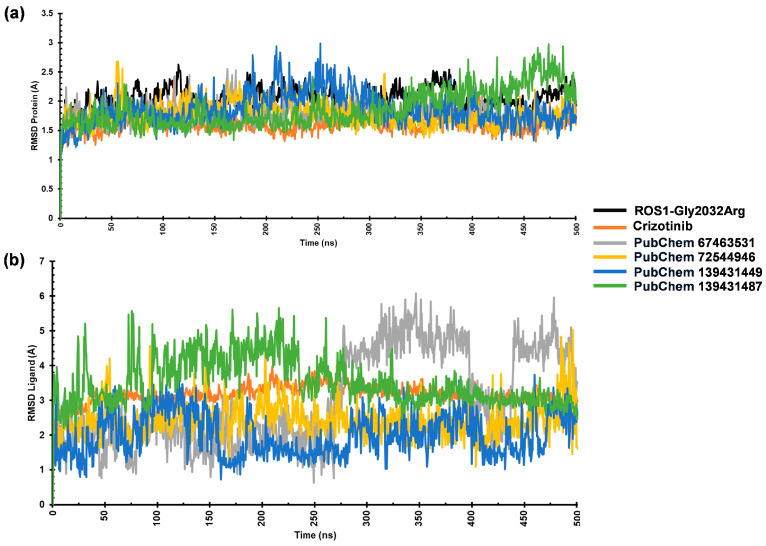
The MD simulation results of ROS1-Gly2032Arg with crizotinib and compounds (**67463531**, **72544946**, **139431449**, and **139431487**). RMSD of (**a**) protein and (**b**) ligands.

The stability of ROS1–ligand complexes was assessed through RMSD analysis during a 500 ns MD simulation. We monitored the RMSD values for both protein and ligand to evaluate structural stability and binding behavior. The average Cα-RMSD values for ROS1-Gly2032Arg and its complexes with the reference, **67463531**, **72544946**, **139431449**, and **139431487** ranged from 1.61 Å to 2.04 Å. The complexes reached equilibrium within the first 50 ns, indicating stable interactions. However, the mutant ROS1 structure exhibited noticeable fluctuations between 60 and 120 ns, which persisted up to 375 ns. Among the studied compounds, the reference crizotinib, **67463531**, and **72544946** demonstrated the lowest RMSD values (~1.61–1.62 Å) (Table 2). On the other hand, the ROS1-**139431449** complex showed slight fluctuations between 175 and 300 ns, peaking around 2.5 Å before stabilizing. These findings suggest that the references **67463531** and **72544946** maintained a stable interaction with ROS1 (Figure 5a). Additionally, we analyzed the ligand RMSD to evaluate binding behavior during the simulation period. The ligand RMSD values for crizotinib, **67463531**, **72544946**, **139431449**, and **139431487** were 3.16 Å, 2.91 Å, 2.41 Å, 2.21 Å, and 3.42 Å, respectively, indicating that ligands maintained structural integrity throughout the simulation. This also confirms their stable interactions within the ROS1 binding site (Figure 5b).

#### 2.4.2. RMSF Analysis

**Figure 6:**
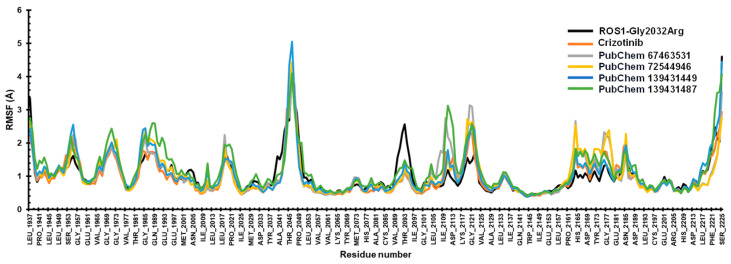
The RMSF of the protein with crizotinib and the screened compounds (**67463531**, **72544946**, **139431449**, and **139431487**).

RMSF analysis helps in identifying the key residues of a protein engaged in interactions with a ligand. The mean RMSF values for the ROS1-mutated protein and the compounds crizotinib, **67463531**, **72544946**, **139431449**, and **139431487** were 1.03 Å, 1.01 Å, 1.01 Å, 1.02 Å, 1.03 Å, and 1.11 Å, respectively. Significant fluctuations were observed in the loop regions of the ROS1-mutant protein, particularly in the Ala2041-Leu2037 (>3 Å), and Val2089-Ile2097 (~3 Å) residues. This can be attributed to the greater susceptibility of the N- and C-termini compared to other regions of the protein. We also noted a consistent trajectory for the compounds, indicating enhanced protein stability (Figure 6).

#### 2.4.3. Contact Map Analysis

**Figure 7:**
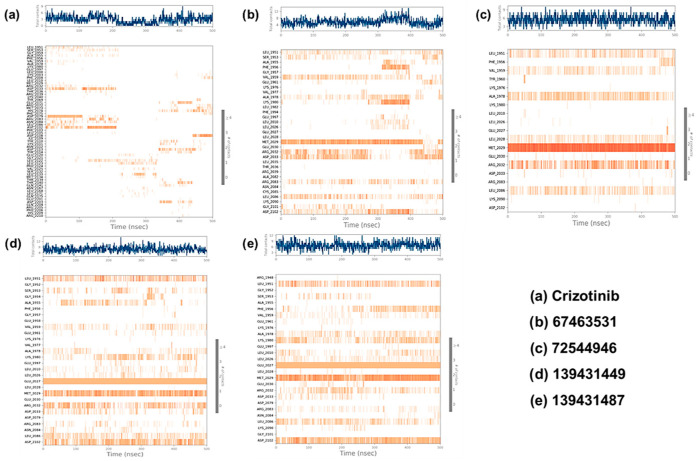
The contact map of ROS1 with crizotinib and compounds (**67463531**, **72544946**, **139431449**, and **139431487**).

**Figure 8:**
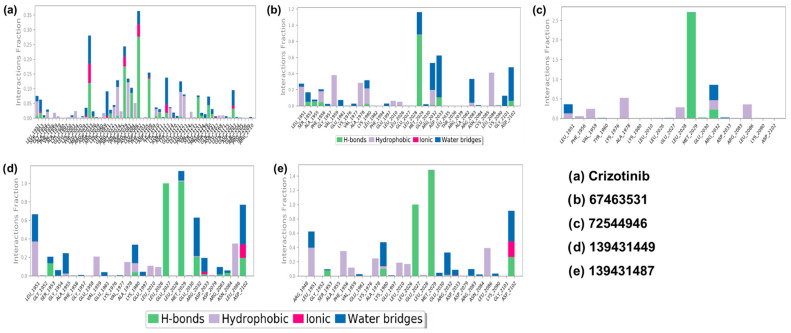
The protein–ligand interaction diagrams for ROS1 with crizotinib and compounds (**67463531**, **72544946**, **139431449**, and **139431487** (**a**–**e**), respectively).

The contact maps for all the complexes were extracted from the simulated trajectory (Figure 7). White or light colors indicate weaker interactions, while darker colors indicate stronger interactions. As can be seen, the protein residues engaged with the ligands via both polar and non-polar non-covalent interactions (Figure 8). Crizotinib exhibits a notable binding profile, forming H-bonding with Ser1953, Arg2032, Asp2033, Glu2071, Met2073, Phe2075, Asp2079, Arg2083, Leu2086, Asp2108, Ile2109, Tyr2110, Asn2112, Gly2121, Ser2132, Leu2133, Met2134, Thr2139, Thr2140, and Gln2201 residues of the binding pocket of ROS1. On the other hand, Leu1951, Ser1953, Gly1954, Phe1959, Glu1997, Lys2003, Leu2010, Lys2040, Arg2072, Met2073, Asp2079, Asn2084, Phe2103, Ile2109, Asp2113, Gly2121, Leu2122, Leu2123, Pro2124, and Glu2201 assisted in forming hydrophobic interactions. Finally, we also noted water bridge interactions involving Leu1951, Ser1953, Leu2000, Leu2010, Met2029, Glu2071, Arg2072, Met2073, Phe2075, Asp2079, Arg2083, Asp2108, Ile2109, Tyr2110, Asn2112, Asp2113, Glu2120, Gly2121, Leu2122, Leu2133, Met2134, Thr2139, Thr2140, Leu2171, Gln2175, Asp2203, and Gln2204. Ionic bonds formed with Ser1953, Met2029, Arg2032, Tyr2069, Phe2075, Leu2105, Asn2112, Tyr2114, Gln2141, and Glu2201 (Figure 8a).

Similarly, in the ROS1-**67463531** complex, H-bonds formed with Ser1953, Ala1955, Ala1978, Leu2028, Glu2030, Arg2032, Ala2082, and Gly2101 residues. Hydrophobic residues include Leu1951, Ala1955, Gly1957, Val1977, Ala1978, Glu1997, Leu2010, Glu2030, Ala2082, and Cys2085. Water bridge interactions involve Leu1951, Ser1953, Ala1955, Phe1956, Val1959, Lys1976, Ala1978, Phe1994, Met2029, Glu2030, Arg2032, Asp2033, Leu2036, Ala2082, Arg2083, Asn2084, Leu2086, Lys2090, Gly2101, and Asp2102 (Figure 8b). Moreover, in the ROS1-**72544946** complex, the interaction pattern includes H-bonds with Leu2028, Met2029, Glu2030, and Arg2032. Hydrophobic residues involved are Leu1951, Phe1956, Val1959, Lys1976, Glu2027, Glu2030, and Arg2083. Water bridge interactions comprise Leu1951, Val1959, Glu2030, and Arg2032 (Figure 8c). In the ROS1-**139431449** complex, the interaction pattern includes H-bonds with Ser1953, Ala1955, Ala1978, Leu2026, Leu2028, Glu2030, Arg2032, Asp2079, Arg2083, and Leu2086. Hydrophobic residues involved are Leu1951, Gly1954, Glu1958, Val1977, Glu1997, Leu2010, Glu2030, and Asn2084. Water bridge interactions comprise Leu1951, Gly1952, Gly1954, Phe1956, Val1959, Lys1976, Ala1978, Glu1997, Leu2010, Leu2028, Glu2030, Arg2032, Asp2033, Asp2079, and Asp2102. Ionic bonds formed with Arg2032, Asp2033, and Leu2086 (Figure 8d). The interaction pattern of the ROS1-**139431487** complex includes H-bonds with Gly1952, Ala1978, Leu2026, Leu2028, Glu2030, Arg2032, Asp2079, and Asp2102. Hydrophobic residues involved are Arg1948, Ala1955, Phe1956, Lys1976, Ala1978, Glu1997, Leu2010, and Asn2084. Water bridge interactions comprise Arg1948, Gly1952, Val1959, Lys1980, Met2029, Glu2030, Arg2032, Asp2079, Arg2083, Leu2086, and Asp2102. Ionic bond formation occurs with Arg2032 and Asp2102 (Figure 8e).

### 2.5. DFT Studies

**Figure 9:**
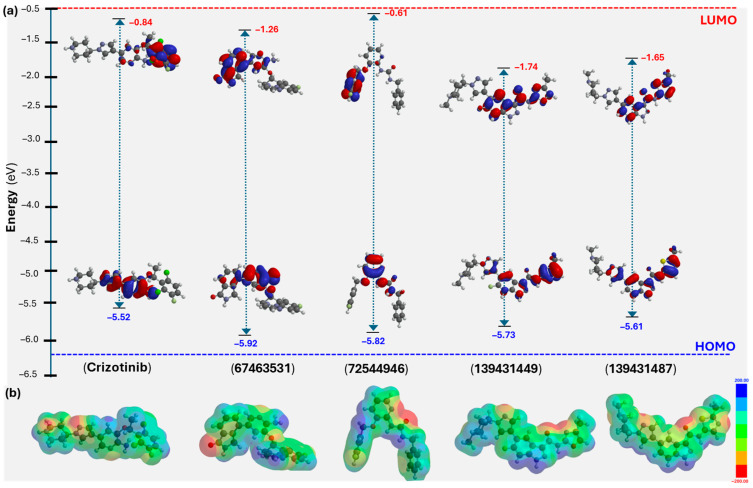
(**a**) Frontier molecular orbitals and (**b**) electrostatic potential map of crizotinib and shortlisted compounds.

Following the molecular docking, ADME/T analyses, and MD studies, DFT calculations at the B3LYP/6-31G* level were performed to explore the molecular features of the identified compounds [30]. While DFT is widely recognized for its accuracy and ability to predict the structure and properties of molecular systems, it has recently attracted interest among researchers for estimating molecular descriptors and correlating them with bioactivity [31,32]. The results of these findings are presented in Table 3 and Figure 9. As shown, the highest occupied molecular orbital (HOMO) is primarily distributed over a common motif, the pyridinyl ring, in both the reference (crizotinib) and the screened compounds (**67463531** and **72544946**, Figure 9a). As expected, the lowest unoccupied molecular orbital (LUMO) was found mainly over the 2,6-dichloro-3-fluorophenyl core in crizotinib. On the other hand, it was over isoindolin-1-one in **67463531** and 3-fluorobenzyl in **72544946** (Figure 9a). Compounds **139431449** and **139431487**, having close structures (only differing in the oxazole/thiazole fragment), showed similar frontier molecular orbital distribution and energy levels. Therefore, in the case of **139431449** and **139431487**, HOMO was delocalized over 6-fluoro-N-(4-methyloxazol-2-yl)-1H-indazole-3-carboxamide and N-(2-methylthiazol-5-yl)-1H-indazole-3-carboxamide, respectively. The HOMO levels for the screened compounds range from −5.52 to −5.92 eV, while the LUMO values vary between –1.74 and −0.84 eV, resulting in ΔE_(H-L)_ values between 3.96 and 4.66 eV. These values were comparable with the reference compounds (ΔE_(H-L)_ = 4.68 eV). Interestingly, we noted a relationship between the HOMO level and the XP GScore of the compounds. The electrostatic potential (ESP) map of the studied compounds is shown in Figure 9b. Different colors of the ESP indicate electron-poor or rich regions. Finally, using the ΔE_(H-L)_ energies, we estimated the chemical reactivity descriptors such as chemical hardness (*η* = 1.98 to 2.34 eV), softness (*σ* = 0.19 to 0.25 eV^−1^), chemical potential (*μ* = −3.73 to −3.18 eV), electrophilicity index (*ω* = 1.97 to 3.48 eV), and electronegativity (*χ* = 3.18 to 3.73 eV) of the compounds.

## 3. Discussion

The ROS1 gene encodes a proto-oncogene tyrosine-protein kinase that plays a role in cellular signalling and oncogenesis [6]. The protein consists of an extracellular domain with nine fibronectin III-like domains and three β-propeller structures, a 23-residue transmembrane segment, and an intracellular tyrosine kinase domain [6,34]. ROS1 has emerged as a significant oncogenic driver in multiple cancers [18]. Targeted therapies, such as crizotinib and lorlatinib, have been developed to inhibit ROS1 fusion proteins, offering effective treatment options for ROS1-positive lung cancer [17,35]. However, resistance mutations, particularly G2032R, present challenges, necessitating the discovery of next-generation inhibitors [36]. G2032R is the most prevalent resistance mechanism in ROS1-rearranged non-small-cell lung cancer (NSCLC), emerging in approximately one-third of patients after crizotinib therapy [37]. Current second-generation inhibitors, such as ceritinib and entrectinib, show limited efficacy against this mutation.

Despite significant progress in computational drug design and the synthesis of ROS1 inhibitors, bypassing resistance remains a challenge. Researchers continue to explore novel scaffolds and adopt optimization strategies to enhance potency, selectivity, and resistance profiles. While promising compounds have been identified [37,38,39], new drug candidates are needed to develop clinically effective ROS1 inhibitors. Recently, Park et al. [40] developed trisubstituted pyrazole-based inhibitors, with some compounds showing low IC_50_ values and strong interactions within ROS1. The inhibitors were tested primarily against wild-type ROS1, leaving their effectiveness against resistant mutations like G2032R unclear. Similarly, Guo et al. [41] focused on developing dual ALK/ROS1 inhibitors to overcome ALK G1202R mutation. They adopted a structure-guided approach, leading to the identification of diarylaminopyrimidine analogues with superior cytotoxicity and nanomolar inhibitory activity against ALK and ROS1. It was suggested that the introduction of heteroatoms, such as sulfur, is beneficial for activity. Drilon et al. [42] studied and identified the ROS1 D2033N mutation as a novel mechanism of acquired resistance to crizotinib in ROS1-rearranged lung cancer. Structural modeling and biochemical assays revealed that this mutation reduces crizotinib binding by altering electrostatic interactions. It was also reported that a heterocyclic small molecule N-(4-((6,7-dimethoxyquinolin-4-yl)oxy)phenyl)-N-(4-fluorophenyl)cyclopropane-1,1-dicarboxamide (cabozantinib) inhibits the mutant form. While the above-mentioned candidates are promising, further clinical studies will be needed to determine their broader applicability and potential off-target effects, which is indeed a time- and money-intensive step.

Considering this, we adopted a comprehensive computational approach to identify new candidates that could bind and inhibit the mutant form of ROS1. Fragment-based molecular docking identified four promising inhibitors (**67463531**, **72544946**, **139431449**, and **139431487**) from 1760 molecules. These top four compounds showed strong binding affinities and favorable pharmacokinetic properties. MD simulations confirmed the stability of ROS1–ligand complexes over 500 ns, especially complexes containing **67463531** and **72544946**. RMSF and contact map analyses highlighted several key residues involved in stable interactions. DFT results also supplement the docking results, as the values of frontier molecular orbital energies and other descriptors for the top two candidates (**67463531** and **72544946**) are close to those of the reference compound, crizotinib.

## 4. Materials and Methods

**Scheme 1:**
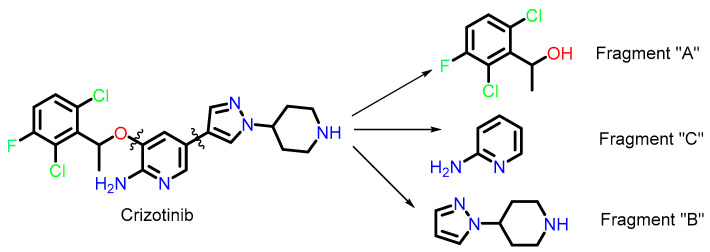
Chemical structures of the fragments used to search the PUBCHEM database.

To collect the maximum diversity of molecules such as crizotinib, we adopted a fragment-based search strategy (Scheme 1). We divided crizotinib into three parts, namely fragments “A” (SMILES: (O[C@@H](C1=C(Cl)C=CC(F)=C1Cl)C), “B” (SMILES: N1(C2CC NCC2)C=CC=N1), and “C” (SMILES: NC1=C(O)C=CC=N1). We then queried these fragment-containing compounds available in the PubChem database [43], leading to the identification of 1760 molecules.

### 4.1. Molecular Docking

#### 4.1.1. Hardware and Software

All computational calculations were conducted on a Dell workstation with 64 GB of RAM with an NVIDIA GeForce RTX 3070 graphics card and 8 GB of GDDR6 memory.

#### 4.1.2. Ligands and Receptor Preparation

A set of 1760 compounds from the PubChem database was obtained in sdf format. The structures were cleaned, energy-minimized, and converted to mae format using Schrödinger Maestro. LigPrep (Schrödinger) was then employed for further optimization and preparation for molecular docking studies [44]. The ROS1 protein consists of 2347 amino acids [6]. Its available structure (PDB ID: 7Z5X) spans residues 1937–2225 but lacks a segment (2104–2123) [24]. To complete the structure, the missing residues were modelled using SWISS-MODEL [45], incorporating the co-crystal ligand from the original structure. Active site residues were identified from UniProt (P08922), crystallographic data, and literature [46]. Further validation was performed using PDBsum and sources in the literature [47].

#### 4.1.3. Pharmacological Properties Predictions

The pharmacological properties (absorption, distribution, metabolism, and toxicity, ADMET) of the compounds under investigation were assessed using the QikProp software V-2024.4 (Schrödinger) [48,49].

#### 4.1.4. Molecular-Docking-Based Virtual Screening

The Glide module is an advanced, three-tiered molecular docking protocol designed to streamline the drug discovery process [50]. A systematic multi-stage virtual screening workflow was employed to identify potential ROS1 inhibitors. Initially, 1760 crizotinib-like compounds were retrieved from the PubChem database and screened using High-Throughput Virtual Screening (HTVS), followed by Standard Precision (SP) and Extra Precision (XP) docking, each retaining the top 50% of ranked molecules. From the XP stage, 25 leading compounds were shortlisted and evaluated [51]. Based on docking performance and key hydrogen bond interactions, four top candidates (PubChem CIDs **67463531**, **72544946**, **139431449**, and **139431487**) were selected. Their binding modes were visualized using PyMOL 3.1, and owing to their favorable binding affinities, interaction stability, and pharmacokinetic profiles, these compounds were advanced for MD simulations [52].

#### 4.1.5. MD Simulations

To predict the stability of the protein–ligand docking complex, MD calculations were conducted using Desmond V4.1 following our previous protocol [53]. The complexes were immersed in a simple point charge (SPC) water model, neutralized with counterions, and adjusted to a physiological salt concentration of 0.15 M [54]. The Particle Mesh Ewald (PME) method was used for electrostatics, with a threshold of 10 for Lennard–Jones interactions. The SHAKE algorithm was employed to constrain the movements of covalent bonds involving hydrogen atoms [54,55]. Initial energy minimization (2000 steps) with solute restraints utilized a hybrid method of steepest descent (10 steps) and the LBFGS algorithm followed by energy minimization (2000 steps) without solute restraints. Simulations at 300 K involved 24 ps in the NPT ensemble with restrained solute non-hydrogen atoms, followed by an additional 24 ps with no constraints. Initial simulations utilized Berendsen thermostats and barostats to monitor temperatures and pressures [56]. Subsequently, a Nose–Hoover thermostat at 300 K and Martyna–Tobias–Klein barostats at 1.01 bar pressure were employed in the NPT ensemble for a relaxed method spanning 500 ns with a time steps of 2 fs [57]. Trajectories (around 20 K) were recorded at a time interval of 4.5 ps.

#### 4.1.6. Density Functional Theory (DFT) Studies

DFT calculations were performed using the Becke three-parameter hybrid functional combined with Lee-Yang-Parr correlation functional (B3LYP) and 6-31G* basis set with the Spartan 24 software. Calculations were performed using the PCM solvation model, and water was selected as the solvent [58,59]. Chemical reactivity descriptors (ionization energy, electron affinity, electronegativity, chemical potential, chemical hardness, softness, and electrophilicity index) were estimated following a previous publication [33].

## 5. Conclusions

The proto-oncogene receptor tyrosine kinase ROS1 plays a pivotal role in regulating cancers, particularly non-small-cell lung cancer (NSCLC). Aberrations such as ROS1 gene fusions have been detected in a subset of NSCLC cases, leading to persistent activation of the ROS1 kinase domain. These mutations are recognized as driver mutations that contribute to unregulated cell growth. Targeting ROS1 with inhibitors like crizotinib has proven effective in treating ROS1-positive NSCLC. In this context, targeting ROS1 presents a promising strategy for developing effective anticancer drugs. A total of 1760 compounds, bearing crizotinib fragments, were retrieved from the PubChem database. This study identified four small-molecule inhibitors, namely **67463531**, **72544946**, **139431449**, and **139431487,** targeting mutated ROS1 with a favorable pharmacokinetic profile. The results of the molecular docking and MD simulations suggest high affinity of these compounds against mutated ROS1. While further in vitro and in vivo validation is required to substantiate these claims, we firmly believe that the compounds are promising and may serve as potential leads for next-generation ROS1 inhibitors.

## Figures and Tables

**Table 1:** The results encompass drug-likeness properties, molecular docking XP Gscore, and MMGBSA calculations for the selected compounds.

**Comp.**	**mol MW**	**RB**	**donorHB**	**accptHB**	**PSA**	**%HOA**	**dipole**	**SASA**	**FOSA**	**FISA**	**PISA**	**WPSA**	**volume**	**RO5**	**XP GScore**	**MM/GBSA**
Crizotinib	450.342	4	3	5.25	73.04	92.19	1.451	698.55	271.34	116.27	190.44	120.49	1283.91	0	−7.50	−55.59
**67463531**	430.44	5	3	6.75	116.82	89.51	5.734	674.27	103.99	173.43	349.88	46.97	1261.85	0	−11.24	−56.10
**72544946**	351.38	6	2	3.75	108.70	80.98	7.484	685.51	74.24	69.80	494.48	46.99	1166.10	0	−10.63	−59.70
**139431449**	423.45	2	1	8	101.03	80.83	6.52	731.04	340.84	139.69	219.99	30.51	1294.20	0	−9.77	−55.31
**139431487**	421.51	2	1	7.5	113.60	77.53	8.51	752.80	339.12	152.58	214.84	46.25	1322.07	0	−9.68	−58.31
**Comp.**	**QPpolrz**	**QPlogPC16**	**QPlogPoct**		**QPlogPw**	**QPlogPo/w**		**QPlogS**	**CIQPlogS**	**QPlogHERG**	**QPPCaco**	**QPlogBB**	**QPPMDCK**	**QPlogKp**	**metab**	**QPlogKhsa**
Crizotinib	44.479	13.294	22.432		12.212	4.143		−5.457	−6.085	−6.114	195.077	−0.142	427.651	−4.67	5	0.781
**67463531**	44.476	13.996	23.792		14.858	3.498		−5.379	−6.625	−5.829	224.514	−1.271	177.99	−3.003	5	0.461
**72544946**	46.457	12.895	21.774		12.839	2.908		−5.162	−4.739	−6.776	116.983	−0.592	79.09	−5.19	3	0.44
**139431449**	47.523	13.79	22.264		12.457	3.255		−5.806	−4.903	−6.942	88.274	−0.744	71.147	−5.445	3	0.612
**139431487**	47.044	14.537	24.652		16.533	2.121		−3.992	−4.198	−7.283	135.829	−0.942	63.25	−4.36	6	−0.111

**Table 2:** The average value of the RMSD of protein and ligand and the RMSF values.

System	RMSD Protein (Å)	RMSD Ligand (Å)	RMSF Protein (Å)
ROS1-Gly2032Arg	2.04	-	1.03
Crizotinib	1.62	3.16	1.01
**67463531**	1.61	2.91	1.01
**72544946**	1.62	2.41	1.02
**139431449**	1.86	2.21	1.03
**139431487**	1.86	3.42	1.11

**Table 3:** DFT (B3LYP/6-31G*)-calculated frontier molecular orbital energies, energy gap (ΔE_(H-L)_), and chemical reactivity descriptors computed from the HOMO-LUMO energies.

Comp.	E_HOMO_ (eV)	E_LUMO_ (eV)	Δ*E* ^a^ (eV)	*I* ^b^ (eV)	*A* ^c^ (eV)	*Χ* ^d^ (eV)	*H* ^e^ (eV)	*σ* ^f^ (eV^−1^)	*μ* ^g^ (eV)	*ω* ^h^ (eV)
Crizotinib	–5.52	–0.84	4.68	5.52	0.84	3.18	2.34	0.21	–3.18	2.16
**67463531**	–5.92	–1.26	4.66	5.92	1.26	3.59	2.33	0.21	–3.59	2.76
**72544946**	–5.82	–0.61	5.21	5.82	0.61	3.21	2.60	0.19	–3.21	1.97
**139431449**	–5.73	–1.74	3.99	5.73	1.74	3.73	1.99	0.25	–3.73	3.48
**139431487**	–5.61	–1.65	3.96	5.61	1.65	3.63	1.98	0.25	–3.63	3.32

^a–h^ for details, see ref [33].

## Data Availability

The original contributions presented in this study are included in the article. Further inquiries can be directed to the corresponding authors.

## Abbreviations

The following abbreviations are used in this manuscript:

ADME	Adsorption, distribution, metabolism, excretion
DFT	Density functional theory
MD	Molecular dynamics
MMGBSA	Molecular Mechanics Generalized Born Surface
NSCLC	Non-small-cell lung cancer
PDB	Protein data bank
RMSD	Root mean square deviation
RMSF	Root mean square fluctuation

## References

[1] Yatabe Y. (2024). Molecular pathology of non-small cell carcinoma. Histopathology.

[2] Gridelli C., Rossi A., Carbone D.P., Guarize J., Karachaliou N., Mok T., Petrella F., Spaggiari L., Rosell R. (2015). Non-small-cell lung cancer. Nat. Rev. Dis. Primers.

[3] Damen M.P., van Rheenen J., Scheele C.L. (2021). Targeting dormant tumor cells to prevent cancer recurrence. FEBS J..

[4] Dagogo-Jack I., Shaw A.T. (2018). Tumour heterogeneity and resistance to cancer therapies. Nat. Rev. Clin. Oncol..

[5] Turner N.C., Reis-Filho J.S. (2012). Genetic heterogeneity and cancer drug resistance. Lancet Oncol..

[6] Roskoski R. (2017). ROS1 protein-tyrosine kinase inhibitors in the treatment of ROS1 fusion protein-driven non-small cell lung cancers. Pharmacol. Res..

[7] Esteban-Villarrubia J., Soto-Castillo J.J., Pozas J., San Román-Gil M., Orejana-Martín I., Torres-Jiménez J., Carrato A., Alonso-Gordoa T., Molina-Cerrillo J. (2020). Tyrosine kinase receptors in oncology. Int. J. Mol. Sci..

[8] Moes-Sosnowska J., Szpechcinski A., Chorostowska-Wynimko J. (2024). Clinical significance of TP53 alterations in advanced NSCLC patients treated with EGFR, ALK and ROS1 tyrosine kinase inhibitors: An update. Tumor Biol..

[9] Davies K.D., Doebele R.C. (2013). Molecular pathways: ROS1 fusion proteins in cancer. Clin. Cancer Res..

[10] Rimkunas V.M., Crosby K.E., Li D., Hu Y., Kelly M.E., Gu T.-L., Mack J.S., Silver M.R., Zhou X., Haack H. (2012). Analysis of receptor tyrosine kinase ROS1-positive tumors in non–small cell lung cancer: Identification of a FIG-ROS1 fusion. Clin. Cancer Res..

[11] Guaitoli G., Bertolini F., Bettelli S., Manfredini S., Maur M., Trudu L., Aramini B., Masciale V., Grisendi G., Dominici M. (2021). Deepening the knowledge of ROS1 rearrangements in non-small cell lung cancer: Diagnosis, treatment, resistance and concomitant alterations. Int. J. Mol. Sci..

[12] Drilon A., Jenkins C., Iyer S., Schoenfeld A., Keddy C., Davare M.A. (2021). ROS1-dependent cancers—Biology, diagnostics and therapeutics. Nat. Rev. Clin. Oncol..

[13] Zhang Y., Zhang X., Zhang R., Xu Q., Yang H., Lizaso A., Xu C., Liu J., Wang W., Ou S.-H.I. (2021). Clinical and molecular factors that impact the efficacy of first-line crizotinib in ROS1-rearranged non-small-cell lung cancer: A large multicenter retrospective study. BMC Med..

[14] Black R.C., Khurshid H. (2015). NSCLC: An update of driver mutations, their role in pathogenesis and clinical significance. Rhode Isl. Med. J..

[15] Roys A., Chang X., Liu Y., Xu X., Wu Y., Zuo D. (2019). Resistance mechanisms and potent-targeted therapies of ROS1-positive lung cancer. Cancer Chemother. Pharmacol..

[16] Michelotti A., de Scordilli M., Bertoli E., De Carlo E., Del Conte A., Bearz A. (2022). NSCLC as the paradigm of precision medicine at its finest: The rise of new druggable molecular targets for advanced disease. Int. J. Mol. Sci..

[17] D’Angelo A., Sobhani N., Chapman R., Bagby S., Bortoletti C., Traversini M., Ferrari K., Voltolini L., Darlow J., Roviello G. (2020). Focus on ROS1-positive non-small cell lung cancer (NSCLC): Crizotinib, resistance mechanisms and the newer generation of targeted therapies. Cancers.

[18] Lin J.J., Shaw A.T. (2017). Recent advances in targeting ROS1 in lung cancer. J. Thorac. Oncol..

[19] Li Y., Lv Y., Zhang C., Fu B., Liu Y., Hu J. (2023). Recent advances in the development of dual ALK/ROS1 inhibitors for non-small cell lung cancer therapy. Eur. J. Med. Chem..

[20] Vanajothi R., Vedagiri H., Al-Ansari M.M., Al-Humaid L.A., Kumpati P. (2022). Pharmacophore based virtual screening, molecular docking and molecular dynamic simulation studies for finding ROS1 kinase inhibitors as potential drug molecules. J. Biomol. Struct. Dyn..

[21] Wu X., Wang Y., Wan S., Zhang J. (2018). Investigation on the binding mechanism of loratinib with the c-ros oncogene 1 (ROS1) receptor tyrosine kinase via molecular dynamics simulation and binding free energy calculations. J. Biomol. Struct. Dyn..

[22] Pathak D., Chadha N., Silakari O. (2016). Identification of non-resistant ROS-1 inhibitors using structure based pharmacophore analysis. J. Mol. Graph. Model..

[23] Hasan S.I. (2025). Exploring the Profiles of ROS1 Tyrosine Kinase: A Structural Analysis of G2032R and D2033N Mutations. Int. J. Appl. Basic Med. Res..

[24] Petrovic D., Scott J.S., Bodnarchuk M.S., Lorthioir O., Boyd S., Hughes G.M., Lane J., Wu A., Hargreaves D., Robinson J. (2022). Virtual screening in the cloud identifies potent and selective ROS1 kinase inhibitors. J. Chem. Inf. Model..

[25] Schwede T., Kopp J., Guex N., Peitsch M.C. (2003). SWISS-MODEL: An automated protein homology-modeling server. Nucleic Acids Res..

[26] Bodade R., Beedkar S., Manwar A., Khobragade C. (2010). Homology modeling and docking study of xanthine oxidase of *Arthrobacter* sp. XL26. Int. J. Biol. Macromol..

[27] Laskowski R.A., MacArthur M.W., Moss D.S., Thornton J.M. (1993). PROCHECK: A program to check the stereochemical quality of protein structures. J. Appl. Crystallogr..

[28] Ntie-Kang F., Nyongbela K.D., Ayimele G.A., Shekfeh S. (2019). “Drug-likeness” properties of natural compounds. Phys. Sci. Rev..

[29] Almasoudi H.H., Nahari M.H. (2025). Targeting Plasmodium falciparum Schizont Egress Antigen-1 in Infected Red Blood Cells: Docking-Based Fingerprinting, Density Functional Theory, Molecular Dynamics Simulations, and Binding Free Energy Analysis. Pharmaceuticals.

[30] Sabe V.T., Ntombela T., Jhamba L.A., Maguire G.E., Govender T., Naicker T., Kruger H.G. (2021). Current trends in computer aided drug design and a highlight of drugs discovered via computational techniques: A review. Eur. J. Med. Chem..

[31] Arias F., Franco-Montalban F., Romero M., Carrión M.D., Camacho M.E. (2021). Synthesis, bioevaluation and docking studies of new imidamide derivatives as nitric oxide synthase inhibitors. Biorg. Med. Chem..

[32] Adekoya O.C., Adekoya G.J., Sadiku E.R., Hamam Y., Ray S.S. (2022). Application of DFT calculations in designing polymer-based drug delivery systems: An overview. Pharmaceutics.

[33] Haque A., Alenezi K.M., Khan M.W.A., Soury R., Khan M.S., Ahamad S., Ahmad S., Gupta D. (2025). In silico evaluation of 4-thiazolidinone-based inhibitors against the receptor for advanced glycation end products (RAGE). J. Biomol. Struct. Dyn..

[34] Charest A. (2015). The ROS1 receptor family. Receptor Tyrosine Kinases: Family and Subfamilies.

[35] Muminovic M., Uribe C.R.C., Alvarez-Pinzon A., Shan K., Raez L.E. (2023). Importance of ROS1 gene fusions in non-small cell lung cancer. Cancer Drug Resist..

[36] Jóri B., Falk M., Hövel I., Weist P., Tiemann M., Heukamp L.C., Griesinger F. (2022). Acquired G2032R resistance mutation in ROS1 to lorlatinib therapy detected with liquid biopsy. Curr. Oncol..

[37] Oesterich L.G., Riess J.W. (2019). ROS1. Targeted Therapies for Lung Cancer.

[38] Cui J.J., Rogers E., Zhai D., Deng W., Huang Z., Whitten J., Li Y. (2016). Ending the endless acquired tyrosine kinase resistance mutations—Design of TPX-0005, a multi-target ALK/ROS1/TRK inhibitor with broad spectrum activity against wild-type and mutants including ALK G1202R, ROS1 G2032R and TRKA G595R. Cancer Res..

[39] Cui J.J., Zhai D., Deng W., Rogers E., Huang Z., Whitten J., Lim J., Li Y. (2018). Abstract B185: TPX-0005, a supreme ROS1 inhibitor, overcomes crizotinib-resistant ROS1 mutations including solvent front mutation G2032R and gatekeeper mutation L2026M. Mol. Cancer Ther..

[40] Park B.S., Al-Sanea M.M., Abdelazem A.Z., Park H.M., Roh E.J., Park H.-M., Yoo K.H., Sim T., Tae J.S., Lee S.H. (2014). Structure-based optimization and biological evaluation of trisubstituted pyrazole as a core structure of potent ROS1 kinase inhibitors. Bioorganic Med. Chem..

[41] Guo M., Zuo D., Zhao T., Li X., Cao J., Qiu Y., Wei S., Zhai X. (2021). Structure-based optimization identified novel furyl-containing 2, 4-diarylaminopyrimidine analogues as ALK/ROS1 dual inhibitors with anti-mutation effects. Eur. J. Med. Chem..

[42] Drilon A., Somwar R., Wagner J.P., Vellore N.A., Eide C.A., Zabriskie M.S., Arcila M.E., Hechtman J.F., Wang L., Smith R.S. (2016). A novel crizotinib-resistant solvent-front mutation responsive to cabozantinib therapy in a patient with ROS1-rearranged lung cancer. Clin. Cancer Res..

[43] Kim S., Chen J., Cheng T., Gindulyte A., He J., He S., Li Q., Shoemaker B.A., Thiessen P.A., Yu B. (2019). PubChem 2019 update: Improved access to chemical data. Nucleic Acids Res..

[44] Sterling T., Irwin J.J. (2015). ZINC 15–ligand discovery for everyone. J. Chem. Inf. Model..

[45] Waterhouse A., Bertoni M., Bienert S., Studer G., Tauriello G., Gumienny R., Heer F.T., de Beer T.A.P., Rempfer C., Bordoli L. (2018). SWISS-MODEL: Homology modelling of protein structures and complexes. Nucleic Acids Res..

[46] Consortium U. (2019). UniProt: A worldwide hub of protein knowledge. Nucleic Acids Res..

[47] Laskowski R.A. (2001). PDBsum: Summaries and analyses of PDB structures. Nucleic Acids Res..

[48] Ioakimidis L., Thoukydidis L., Mirza A., Naeem S., Reynisson J. (2008). Benchmarking the reliability of QikProp. Correlation between experimental and predicted values. QSAR Comb. Sci..

[49] Ahamad S., Junaid I.T., Gupta D. (2024). Computational design of novel tau-tubulin kinase 1 inhibitors for neurodegenerative diseases. Pharmaceuticals.

[50] Sandor M., Kiss R., Keseruű G.r.M. (2010). Virtual fragment docking by Glide: A validation study on 190 protein−fragment complexes. J. Chem. Inf. Model..

[51] Pagadala N.S., Syed K., Tuszynski J. (2017). Software for molecular docking: A review. Biophys. Rev..

[52] DeLano W.L. (2002). Pymol: An open-source molecular graphics tool. CCP4 Newsl. Protein Crystallogr..

[53] Desmond S. (2021). Schrödinger. https://www.schrodinger.com/desmond.

[54] Petersen H.G. (1995). Accuracy and efficiency of the particle mesh Ewald method. J. Chem. Phys..

[55] Steinbrecher T., Mobley D.L., Case D.A. (2007). Nonlinear scaling schemes for Lennard-Jones interactions in free energy calculations. J. Chem. Phys..

[56] Ke Q., Gong X., Liao S., Duan C., Li L. (2022). Effects of thermostats/barostats on physical properties of liquids by molecular dynamics simulations. J. Mol. Liq..

[57] Martyna G.J., Tobias D.J., Klein M.L. (1994). Constant pressure molecular dynamics algorithms. J. Chem. Phys..

[58] Mennucci B., Tomasi J., Cammi R., Cheeseman J.R., Frisch M.J., Devlin F.J., Gabriel S., Stephens P.J. (2002). Polarizable continuum model (PCM) calculations of solvent effects on optical rotations of chiral molecules. J. Phys. Chem. A.

[59] Kruse S.J., Forbes T.Z., MacGillivray L.R. (2025). Effects of Ionizing Radiation on Halogen-Bonded Dipyridyl-Naphthalenediimide Cocrystals. Cryst. Growth Des..

